# Implementation of the WHO safe childbirth checklist program at a tertiary care setting in Sri Lanka: a developing country experience

**DOI:** 10.1186/s12884-015-0436-0

**Published:** 2015-02-04

**Authors:** Malitha Patabendige, Hemantha Senanayake

**Affiliations:** University Obstetrics Unit, De Soysa Hospital for Women, Colombo, Sri Lanka; Faculty of Medicine, University of Colombo, Colombo, Sri Lanka

**Keywords:** Safe childbirth, Checklist, World health organization, Sri Lanka

## Abstract

**Background:**

To study institutionalization of the World Health Organization’s Safe Childbirth Checklist (SCC) in a tertiary care center in Sri Lanka.

**Method:**

A hospital-based, prospective observational study was conducted in the De Soysa Hospital for Women, Colombo, Sri Lanka. Healthcare workers were educated regarding the SCC, which was to be used for each woman admitted to the labor room during the study period. A qualitatively pretested, self-administered questionnaire was given to all nursing and midwifery staff to assess knowledge and attitudes towards the checklist. Each item of the SCC was reviewed for adherence.

**Results:**

A total of 824 births in which the checklist used were studied. There were a total of births 1800 during the period, giving an adoption rate of 45.8%. Out of the 170 health workers in the hospital (nurses, midwives and nurse midwives) 98 answered the questionnaire (response rate = 57.6%). The average number of childbirth practices checked in the checklist was 21 out of 29 (95% CI 20.2, 21.3). Educating the mother to seek help during labor, after delivery and after discharge from hospital, seeking an assistant during labor, early breast-feeding, maternal HIV infection and discussing contraceptive options were checked least often. The mean level of knowledge on the checklist among health workers was 60.1% (95% CI 57.2, 63.1). Attitudes for acceptance of using the checklist were satisfactory. Average adherence to checklist practices was 71.3%. Sixty eight (69.4%) agreed that the Checklist stimulates inter-personal communication and teamwork. Increased workload, poor enthusiasm of health workers towards new additions to their routine schedule and level of user-friendliness of Checklist were limitations to its greater use.

**Conclusions:**

Amongst users, the attitude towards the checklist was satisfactory. Adoption rate amongst all workers was 45.8% and knowledge regarding the checklist was 60.1%. These two factors are probably linked. Therefore prior to introducing it to a facility awareness about the value and correct use of the SCC needs to be increased, while giving attention to satisfactory staffing levels.

## Background

Poor quality care during institutional births in low resource settings is one of the main contributing factors towards preventable childbirth-related harm to the mother and neonate. Following on the success of the World Health Organization (WHO) surgical safety checklist, it has developed the Safe Childbirth Checklist (SCC), a 29-item tool that targets major global causes of maternal and neonatal morbidity and mortality [1]. It aims to address issues of poor quality of care that could be effectively addressed through the use of a checklist. This is an intervention analogous to the pre-flight checklist used in the aviation industry where Pilots are alerted to unanticipated deficiencies and malfunctions in the tools required for safety.

The 29 items of the SCC addresses major causes of maternal mortality (e.g. post-partum haemorrhage, infection, and obstructed labour and hypertensive disorders), intrapartum stillbirths (e.g. poor intrapartum care) and neonatal deaths (e.g. birth asphyxia, infection and complications related to prematurity) [1]. It has been tested for usability in ten countries across Africa and Asia [1]. In 2010, 287 000 women died during pregnancy and childbirth and 2.6 million stillbirths occurred worldwide [1]. Most of these were reported from developing countries, the majority of which are preventable [1].

With the WHO Surgical Safety Checklist [2], it has been found that its implementation was associated with concomitant reductions in the rates of deaths and complications among patients undergoing non-cardiac surgeries [3]. Pilot testing of the SCC in South India has demonstrated a significant improvement in the delivery of essential safety practices by healthcare workers after its introduction [4,5].

Sri Lanka is a country that has achieved significant reductions in maternal and infant mortality rates with cost-effective interventions, to reach a preeminent status in maternal and childcare in the developing world [6,7]. Sri Lanka’s, Maternal Mortality Rate was 37.7 per 100,000 live births in 2012 and the Neonatal Mortality Rate 6 per 1000 live births [8-10]. The decline in maternal death rates however has been negligible in the past two decades [8]. To improve this situation, quality of care will require to be reinforced and the SCC is potentially a tool that could help this aspect. Since Sri Lanka has achieved a good level of spread of emergency and non-emergency facilities with staff who believe they already provide high quality care, introduction of a new tool may pose unique challenges.

The main objective of this study was to implement the SCC in the setting of a tertiary care hospital in Sri Lanka. The De Soysa Hospital for Women (DSHW) is a major tertiary maternity care hospital in Sri Lanka with referrals from the whole country and well-trained staff comprising obstetricians, neonatologists, physicians, other junior doctors, nurses, nurse-midwives and midwives. It has an average of 800 births per month. It would be reasonable to surmise that the results of implementation of the SCC in such a setting may prove to be different to those from other countries.

This project was a part of the “WHO Safe Childbirth Checklist Collaboration”, which aims to contribute to understanding the best conditions for implementation of the checklist.

The level of implementation, acceptance and adherence to the each item of the SCC among care providers were to be studied.

## Methods

A hospital-based, prospective observational study was carried out in the obstetric wards of De Soysa Hospital for Women (DSHW), Colombo, Sri Lanka in November and December 2013. The investigators initially gave the necessary basic education regarding the SCC to healthcare workers in the hospital, covering an introduction, its importance, patient safety, components of SSC, how to and when to it. The healthcare workers included were nurses, nurse-midwives and midwives involved in care during labor. Authors reinforced the education by repeated visits to the wards and by giving further instructions orally and using printed leaflets. Soon after the education was completed, the SCC was introduced. SCC were attached to clinical notes of every woman who entered the labor room. This was kept with the mother’s notes until discharge at which point it was collected by the researchers and compliance to each of the 29 items was studied.

Each pregnant woman entering a labor room during the study period and all the nursing and midwifery staff working in labor rooms and postnatal wards during the study period in DSHW were included in the study. Women who underwent elective caesarean sections without entering the labor rooms were excluded.

At the end of the study period, a self-administered pretested anonymous questionnaire consisting of three sections was administered to all eligible staff. Pre-testing was done with a focus group discussion with a group of nurses and midwives (n = 10) working in a labor ward at the Teaching Hospital, Mahamodara, Galle, Sri Lanka, a Teaching Hospital not included to this study. This questionnaire consisted of three sections: Section 01 inquired about basic characteristics of participating health workers; In Section 02, the level of knowledge regarding SCC and knowledge regarding SCC was assessed by 05 questions and a score given as a percentage. These 05 questions were targeting components of SCC and its usage, considering a basic understanding of their level of knowledge about the SCC; In Section-03, a five-point Likert scale for five stems assessed attitudes towards acceptance of the SCC.

Data analysis was carried out using standard statistical methods. Checklist details from all checklist forms and questionnaires were entered into data sheet and descriptive statistics were used to summarize the data. Measures of dispersion and 95% confidence intervals were calculated.

Ethical aspects of the study were reviewed by the Ethical Review Committee of the (EC-13-151), Faculty of Medicine, University of Colombo, Sri Lanka, which granted approval for the study to be conducted.

## Results

Out of a total of 1800 births during the study period there were 824 live births in whom the SCC was used, giving an adoption rate of 45.8%. There were approximately 170 nursing and midwifery staff who were eligible, out of whom completed questionnaires were received only from 98 with a response rate of 57.6%.

Basic characteristics of nurses and midwives (n = 98) are shown in Table 1. Average childbirth practices checked in the SCC were 21 out of 29 (95% CI 20.2; 21.3 median (IQR) of 25 (9.7), mode 28; range 0 to 29). Table 2 shows adherence with each of the 29 items in the SCC. Average adherence to checklist practices was 71.3% as calculated by using adherence rates to each of the 29 items in SCC presented in Table 2. As in Table 2, checklist item numbers 08, 13, 21, 22, 27, 28, and 29 were checked least often (less than in 70%). The rate for the rest of the criteria was above 70%. Educating mother/companion to seek help (if needed or danger signs appear) during labor, after delivery and after discharge from hospital, seeking help or assistance from other staff during labor, early commencement of breastfeeding, management of maternal HIV and discussing family planning options with the mother were those checked least often. The 27^th^ item, which is regarding maternal HIV, was checked with the least frequency (124, 15% of cases). Checklist item on discussing family planning options to mother was checked in 56.4% of cases.

**Table 1:** **General characteristics of the participated health workers**

**Participated health workers**	**Mean age (years) (95% CI)**	**Average years of experience in an obstetric ward (95% CI)**	**Average knowledge score (%)(95% CI)**
**Nurses (n = 28)**	29.3(28.6,30.0)	5.4(3.0,7.8)	55.7(49.1,62.3)
**Nurse-midwives (n = 49)**	33.4(32.1,34.8)	5.4(4.3,6.5)	63.1(59.2,67.0)
**Midwives (n = 21)**	33.9(31.0,36.8)	5.5(3.3,7.8)	59.1(53.3,64.8)

**Table 2:** **Adherence to each 29 items in WHO safe childbirth checklist**

**Checklist item in WHO SCC**	**Frequency of checking of each item (%) n = 824**
***On admission***	
1. Does mother need referral?	687(83.4)
2. Partograph started?	691(83.9)
3. Does mother need to start antibiotics?	688(83.5)
4. Does mother need to start magnesium sulfate?	688(83.5)
5. Does mother need to start Nevirapine?	682(82.8)
6. Encourage birth companion to be present at birth	609(73.9)
7. Confirm supplies available to clean hands and wear gloves for each vaginal exam	644(78.2)
8. Confirm that Mother or Companion will call for help during labor if needed	519(63)
***Just before pushing (or before Cesarean)***	
9. Does mother need to start antibiotics?	637(77.3)
10. Does mother need to start magnesium sulfate?	632(76.7)
11. Confirm essential supplies are at bedside for mother	593(72)
12. Confirm essential supplies are at bedside for baby?	593(72)
13. Assistant identified and ready to help at birth if needed?	442(53.6)
***Soon after birth (within 1 hour)***	
14. Is mother bleeding abnormally?	619(75.1)
15. Does mother need to start antibiotics?	615(74.6)
16. Does mother need to start magnesium sulfate?	607(73.7)
17. Does baby need referral?	615(74.6)
18. Does baby need antibiotics?	611(74.2)
19. Does baby need special care/monitoring?	611(74.2)
20. Does baby need Antiretrovirals?	603(73.2)
21. Started breastfeeding and skin-to-skin contact?	566(68.7)
22. Confirm that mother/companion will call for help if danger signs present	449(54.5)
***Before discharge***	
23. Is Mother’s bleeding controlled?	657(79.7)
24. Mother to start antibiotics?	658(79.9)
25. Babyto start antibiotics?	660(80.1)
26. Is Baby feeding well?	653(79.2)
27. If Mother HIV positive, Mother and Baby have ARVs for 6 weeks?	124(15)
28. Discuss and offer family planning options to Mother	465(56.4)
29. Arrange follow-up and confirm Mother/Companion will seek help if danger signs are present after discharge	407(49.4)

WHO SCC-World Health Organization Safe Childbirth Checklist.

Mean level of knowledge on WHO SCC was 60.1% (95% CI 57.2, 63.1) among all health workers. As indicated in Table 3, attitudes for acceptance of using WHO SCC among health workers were high. Further, 68 (69.4%) out of 98 agreed that WHO SCC stimulates inter-personal communication and teamwork among nurses, midwives and doctors.

**Table 3:** **Attitudes for acceptance of using WHO SCC in routine practice among participated health workers**

**Attitudes for acceptance of using WHO SCC in routine practice**	**Nurses (n = 28)**	**Nurse-midwives (n = 49)**	**Midwives (n = 21)**	**Total (n = 98)**
**1. Implementing the WHO SSC is a good decision. (Strongly agree or Agree)**	28(100)	49(100)	21(100)	98(100)
**2. WHO SCC stimulates inter-personal communication and team work among nurses, midwives and doctors. (Strongly agree or Agree)**	24(85.7)	29(59.2)	15(71.4)	68(69.4)
**3. WHO SCC helps to minimize errors. (Strongly agree or Agree)**	28(100)	46(93.9)	21(100)	95(96.9)
**4. If you or your closest family member is undergoing childbirth and this WHO SCC is being used for your/her childbirth. What is your opinion? (Strongly agree or Agree)**	28(100)	47(95.9)	19(90.5)	94(95.9)
**5. Using the WHO SCC in Sri Lanka is practical. (Strongly agree or Agree)**	27(96.4)	45(91.8)	20(95.2)	92(93.9)

WHO SCC-World Health Organization Safe Childbirth Checklist.

## Discussion

In a pilot test conducted in South India in 2010, essential childbirth-related care practices improved from an average of 10 of 29 practices at baseline to an average of 25 after the introduction of a training programme related to the SCC [4].

In our study, the adoption rate of SSC was 45.8% and the response rate 57.6%. Average childbirth practices checked as per WHO SCC in our study were 21 out of 29 and average adherence to checklist practices was 71.3%. According to Table 3, attitudes for acceptance of using SCC are satisfactory and most of the responses scored above 90%, indicating a high acceptance rate for the SCC in the DSHW. These adherence and adoption rates could be considered the baseline figures in this tertiary care hospital.

In Table 3, the fact that a high percentage of the health workers would want it used in case the woman delivering was herself or a family member can be considered as a sign of high acceptance. Lack of motivation and poor enthusiasm of health workers towards new introductions to their routine schedule, increased work load, lack of staff in the hospital and level of user friendliness of the SCC were the main barriers to its greater usage. It would be interesting to explore deeper into the fact that we implemented it in a tertiary care hospital that is different to where the pilot study in India [4] was undertaken. Findings could differ between sites, depending on the complexity of the cases, levels of staffing and skill levels of different cadres. It was possible staff in DSHW with their high self-confidence may have felt that they did not require a checklist to perform their duties. These understandings could enhance its contribution to the field.

As shown in Table 3, 68 (69.4%) agreed that the SCC could stimulate inter-personal communication and teamwork among nurses, midwives and doctors. This is encouraging, since at the time of conducting this study there was a major controversy involving the nurses and midwives regarding the boundaries of their duties. This was in operation both at national and local institutional levels.

Educating to seek help during labor, after delivery and after discharge from hospital, seeking an assistant during labor was not checked satisfactorily. Checking the item on encouraging a birth companion to be present was 73.9%. However, as we noted only a very few mothers were keeping a birth companions despite this encouragement. Some of the wards involved in this study had only recently introduced the concept of allowing a labor companion. Checklist item on discussing family planning options has been checked only in 56.4%. The latest available national contraceptive prevalence is 68% [9,10]. Any institutional delivery in which this aspect is not discussed must be considered as a lost opportunity. The SCC has highlighted this important failure. However, this may have been due to the staff considering family planning as something that is best discussed either during pregnancy or in the postnatal period, which is the usual practice in Sri Lanka. The checklist item regarding maternal HIV was the item checked with the least frequency (15%). This may be a reflection of the staff taking this aspect for granted, since Sri Lanka is a ‘low-prevalence country’ for HIV [10]. In fact, none of these 124 cases were HIV positive in DSHW.

It would have been useful if the education regarding the checklist was given to all health care providers including medical personnel. Educating the medical professionals would have possibly had a positive impact on the uptake of the SCC, with their leadership role in DSHW. In these hierarchical environments, everyone needs to be encouraging use of these tools even if it is the primary responsibility of the nurse, midwife and nurse-midwife to complete it. As a suggestion for future implementation procedures, it is worthwhile to educate and address all the hierarchical positions in health care team, removing another obstacle to its implementation.

In Sri Lanka more than 99% births are hospital-based institutional deliveries, skilled birth attendants are available [7,10]. Skilled attendance at births is considered to be the single most critical intervention for ensuring a safe childbirth [11,12]. UNFPA has initiated using the term “midwives and others with midwifery skills” (MOMS), to define a “skilled birth attendant”. Also it defines a midwife in a broader context to include nurses, physicians and others who have obtained proficiency in midwifery skills [11]. Evidence from many countries, including Sri Lanka indicates that skilled midwives functioning in the community from preconception onwards can have a drastic impact on reduction of maternal and neonatal mortality and morbidity [12,13]. In WHO recommendations to improve maternal and newborn health, there are specific roles related to childbirth assigned to each health worker in the team [14]. Teamwork is very important in care during childbirth. Almost all respondents agreed that the SCC would help to minimize errors. Checklists are the way the aviation industry circumvents problems that may result from dependence on the judgment and memory of individuals. The same principle could help save the lives of mothers and their babies.

Identification of barriers is necessary to achieve a better uptake of interventions and to improve implementation of clinical practice strategies [15]. Also, discussions of clinical practice problems with health workers are known to help improve care quality and adherence to clinical guidelines [16]. Behavioral interventions are known to be effective, but it is always challenging to bring evidence based changes into routine practice [15,17,18]. Therefore before and after implementing this kind of programme proper training and regular surveillance is worthwhile to maintain optimal use of the intervention. Except for the items on referrals and starting the partogram, rest of the items in SCC are very practical and helpful to healthcare workers to achieve safe practice. Without the SCC, it may be difficult to cover all these aspects. Also we could suggest to the SCC Collaboration to consider numbering each of the items in the checklist improving its user-friendliness. Currently in centers providing care for laboring mothers, delivery practices are carried out based on practical knowledge of the care providers, not according to checklist-based procedures. Therefore, especially in centers with a higher workload as in developing countries where the chances of errors are higher, implementation of checklist-based practices will obviously increase the safety and improve quality.

Evidence from our study should be interpreted in the context of its limitations. Checking of SCC items was not directly observed as in the pilot test in South India [4]. Perception on teamwork is difficult to be assessed with a questionnaire and the self-administered nature of it could also be considered as a limitation. We used a purely descriptive format and there was no comparison group. A follow-up study could see if the practices with the checklist improved over time and if there is a sustained impact.

## Conclusions

Adoption of the WHO Safe Childbirth Checklist is poor in this tertiary care hospital in Sri Lanka where a good level of coverage of maternity care exists. However, healthcare workers’ attitudes towards acceptance of using the SCC to reduce maternal and neonatal childbirth-related harm were positive. The gap between and adoption rate and level of knowledge on SSC needs to be addressed before attempting to implement the SCC in a new setting. Educating all categories of staff, including those who may have a supervisory role is likely to have a positive impact. This study opens the discussion on factors that could lead to improved use of the SCC.
